# Application of Ground-Penetrating Radar for Detecting Internal Anomalies in Tree Trunks with Irregular Contours

**DOI:** 10.3390/s18020649

**Published:** 2018-02-22

**Authors:** Weilin Li, Jian Wen, Zhongliang Xiao, Shengxia Xu

**Affiliations:** School of Technology, Beijing Forestry University, Beijing 100083, China; liweilin@bjfu.edu.com (W.L.); xzliang@bjfu.edu.cn (Z.X.); xushengxia@bjfu.edu.cn (S.X.)

**Keywords:** ground-penetrating radar (GPR), non-destructive testing, tree trunk, internal structure estimation

## Abstract

To assess the health conditions of tree trunks, it is necessary to estimate the layers and anomalies of their internal structure. The main objective of this paper is to investigate the internal part of tree trunks considering their irregular contour. In this respect, we used ground penetrating radar (GPR) for non-invasive detection of defects and deteriorations in living trees trunks. The Hilbert transform algorithm and the reflection amplitudes were used to estimate the relative dielectric constant. The point cloud data technique was applied as well to extract the irregular contours of trunks. The feasibility and accuracy of the methods were examined through numerical simulations, laboratory and field measurements. The results demonstrated that the applied methodology allowed for accurate characterizations of the internal inhomogeneity. Furthermore, the point cloud technique resolved the trunk well by providing high-precision coordinate information. This study also demonstrated that cross-section tomography provided images with high resolution and accuracy. These integrated techniques thus proved to be promising for observing tree trunks and other cylindrical objects. The applied approaches offer a great promise for future 3D reconstruction of tomographic images with radar wave.

## 1. Introduction

The typical healthy trunk is a multilayer structure. It commonly has three sub-layers: the heartwood layer in the center, the active sapwood layer, the bark of living phloem and a dead cork layer [1]. Usually, the variation of water content in different parts results in dielectric constant differences, while the ion concentration in the trunk is relevant to its electrical conductivity. Trees are constantly endangered by natural defects and biological degradation and it is highly important to prevent collapses of trees around roads. Trees with internal anomalies such as knots, decay, and cracks may be at the risk of falling down, which is of great concern. Therefore, it is of great importance to detect the defects of tree trunks rapidly and accurately.

Conventionally, the anomalies of tree trunks in the field are assessed by core drilling methods. In order to avoid destruction of valuable trees, non-invasive methods are widely used for the evaluation of the state of the trunk [2]. Ultrasonic tomography and stress waves used for detecting internal defects have the disadvantage of being time-consuming and lacking the desired accuracy [3]. In recent years, ground penetrating radar (GPR), which allows for rapid and real-time measurements, has received great attention for nondestructive testing (NDT). This technique has been applied for pavement investigation [4], geological exploration [5], civil engineering [6], subsurface archaeological investigation [7] and thin-layer thickness estimation [8]. In the forestry field, GPR has provided an important method of root system architecture reconstruction [9], root diameter or biomass estimation [10], and moisture content [11]. However, interpretation of raw GPR radargrams is difficult because of the complex geometrical information of tree trunks, the coupling of layers and signal attenuation.

Currently, most studies of the tree trunk nondestructive detection by GPR were concentrated on the estimation of internal defects. Butnor et al. [12] compared measurements of decay from stem cross sections and increment cores for three conifer species (*Pseudotsuga menziesii*, *Thuja plicata* and *Tsuga heterophylla*), and found that near-surface decay, air-filled voids and desiccated boles had unique electromagnetic signatures, which could be separated from other defects. GPR successfully estimated the percent area of air-filled cavities and was not significantly different than results from destructive sampling. Udaya et al. [13] made use of GPR to detect the position of internal defects of trees. In [14] a ray-based tomography method using GPR to reconstruct the internal structure of the trunk cross-section of a living oak tree was presented. It was difficult to construct a unified test model due to high moisture content, which varies significantly in different species under different conditions [15]. The dielectric properties of trunk layers could be estimated invasively or non-invasively. Invasive techniques are not suitable for the living trees, while the non-invasive estimation based on the reflected GPR signals is treated here. Lv et al. [16] tested four typical trees trunks’ (polar, willow, pine, eucalyptus) moisture content and dielectric constant under radar wave frequency, respectively, and established models of the relationship between moisture content and dielectric constant of the trees trunks. Reci et al. [17] carried out to study how the GPR signal is affected by moisture variation in wood materials, and investigated the effects of the wood fiber direction with respect to the polarisation of the electromagnetic field. Martínez-Sala et al. [18] assessed the dielectric anisotropy in timber using the nondestructive GPR technique. Ježová et al. [19] investigated particularities of tree trunks radar images, considering the circumferential data acquisition geometry, and described the reflection curve gained from a cylindrical medium. In [20] GPR was used to detect the cavity cracking of Masson pine, and the results indicated that the moisture content of timber has an impact on the radar detection results and that the estimated boundary of the cavity damages may be offset slightly. Despite extensive applications of non-invasive methods for subsurface characterizations, the potential of these techniques for accurate detection of the internal defects in tree trunks has not been fully explored.

The objective of this study was to detect particular features occurring in tree trunks due to their irregular contours using GPR. We used the Hilbert transform algorithm and the reflection amplitudes to estimate the relative dielectric constant. Numerical simulations, laboratory and field experiments were carried out to corroborate the applied methodology. For numerical simulations, we employed gprMax2D in [21] which solves two dimensional (2D) Maxwell equations using the Finite-Difference Time-Domain (FDTD) technique [22]. Furthermore, we analytically characterized the shape of a reflection curve of a circular inhomogeneity hidden in the trunk for determination of the location, shape and size of anomalies. Moreover, the results of cross-section tomography were compared with the TRU analysis software (TreeWin) [23].

## 2. Materials and Methods

### 2.1. GPR Detection Principle

**Figure 1:**
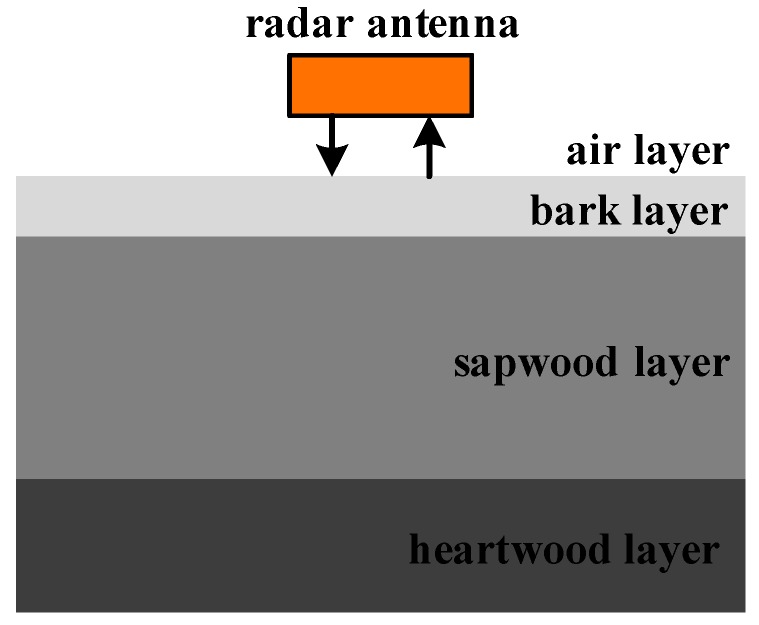
The sketch map of GPR detection.

The principle of GPR detection is to send high-frequency electromagnetic waves into the skin of trees through the antenna, and electromagnetic waves propagate inside trees. GPR research is based on the classical Maxwell equations. As shown in Figure 1, there are differences in dielectric constants at the juncture of normal structural layers or cavities, decay and other defects, which result in electromagnetic wave reflection. The receiving antenna receives the echo signal. The internal structure and defects of the trunk can be analyzed according to the radar echo waveform, intensity and time [24].

The amplitude of the reflected echo mainly depends on the electrical difference between the dielectric layers. The reflected signal gets strong as the electrical difference increases [25]. The characteristic of reflected waves is obvious through the heartwood layer in the center, the active sapwood layer, the bark of living phloem and dead cork layer.

The research collects the data with the TRU™ Tree Radar System (TreeRadar Inc., Silver Spring, MD, USA), which contains the coupling antenna radar centered on 900 MHz and the SIR series data collector. The analysis routine is developed in order to predict internal structure of tree trunk better.

### 2.2. Layer Localization Method

Huang et al. [26] proposed a new time-frequency analysis method (Hilbert-Huang Transform). The method for the analysis of non-stationary and nonlinear signal is more intuitive and adaptable than the threshold detector and the matched filter detector. In theory, the signal is processed by empirical mode decomposition (EMD). Then we get a finite number of intrinsic mode components (IMF) and a residual signal that represents a signal change trend. Each IMF obtained by using Hilbert transform time is put forward for frequency analysis [27].

According to Rosenfeld sub-band product theory, if the noise signal can be decomposed into a plurality of frequency sub-band components, each component of the point can highlight the useful signal and eliminate the noise by dot product. Based on this theory, Kim et al. [28] proposes wavelet detector to detect biomedical signals at a low signal to noise ratio (SNR). Inspired by the wavelet detector, in this paper, IMF component is used to construct the detection algorithm. The first step of the Hilbert algorithm is to process the echo data by EMD and obtain the corresponding N IMF c_1_(t)~c_N_(t). Then the dot product of the absolute value of all components IMF is calculated:

(1) $$P\left(t\right)=\left|\overset{N}{\underset{i=1}{\prod }}c_{i}\left(t\right)\right|$$

The received signal is composed of the multi-peak pulses because the echo signal contains the positive and negative peaks, which easily increases the false alarm rate and deteriorate the detection rate. The signal needs to be smoothed by the window function. Convolution using P(t) and W(t) window function is used to complete smoothing:

(2) T(t)=W(t)⊗P(t)

The final step is to compare T(t) to the threshold V(t). If the T(t) exceeds the threshold, take the time-delay of the maximum value as the time-delay of the detected pulse. Otherwise, there are no detected targets.

**Figure 2:**
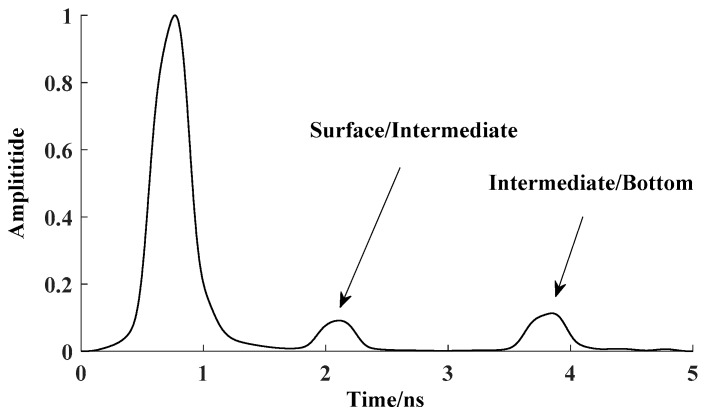
The detected layers of single-channel wave using Hilbert algorithm.

As shown in Figure 2, the Hilbert filter method is adapted to analyze the signal channel echo data of the forward model. The reflected signal of the first layer, the second layer and the third layer extracted from the echo signal is presented. Hilbert product algorithm is verified to be able to locate different media layers.

### 2.3. Estimation of Relative Dielectric Constant from Reflection Amplitudes

Tree trunk layers are typically composed of various materials other than healthy wood, such as air-voids, decay zones, etc. The dielectric properties of trunk layers would vary depending on the structures inside, which may be greatly affected by varieties of trees, environmental conditions, external factors, etc. Previous studies demonstrated that tree trunks have relative dielectric constants ranging between 5 and 13 [12,29,30]. As a consequence, the dielectric properties of trunk layers are usually unknown and difficult to predict. Furthermore, the relative dielectric constant of a layer is needed to determine its thickness by estimating the speed of the electromagnetic wave and by measuring its travel time between two layer interfaces. 

The amplitudes of the reflected pulses depend on the contrast of the relative dielectric constants of the adjacent layers. Given A_m_ as a relative reflection amplitudes for layer m (the ratio between amplitudes of the reflected and the incident signals), the relative dielectric constants of the first (ε1) and second (ε2) trunk layers are given by:

(3) ε1=(1−A1Am1+A1Am)2

(4) ε2=ε1[1−(A1Am)2−A2Am1−(A1Am)2+A2Am]2

The relative dielectric constant of the second trunk layer depends on the relative dielectric constant ε_1_. Similarly, the relative dielectric constant of the third layer ε_3_ depends on the dielectric properties of the two layers above it. Consequently, the relative dielectric constant of a layer depends on the estimated relative dielectric constants of the layers above it. The error of estimating the relative dielectric constant of a layer based on the GPR reflected pulses amplitudes would increase with the depth of the layer.

### 2.4. Trunk Contour Acquisition

The 3D structure modeling methods of trees are mainly based on the rules of plant growth, the images, and sketches [31]. Furthermore, the point cloud data represented by 3D laser scanning technology, which can measure point cloud data of trees quickly and effectively, has also become one of the important methods to get the 3D structure of trees in recent years [32]. Moreover, the 3D visualization of these techniques has been realized by [33]. A point cloud is a set of data points in a coordinate system. In a 3D coordinate system, these points are usually defined by *X*, *Y* and *Z* coordinates. They are often intended to represent the external surface of an object.

**Figure 3:**
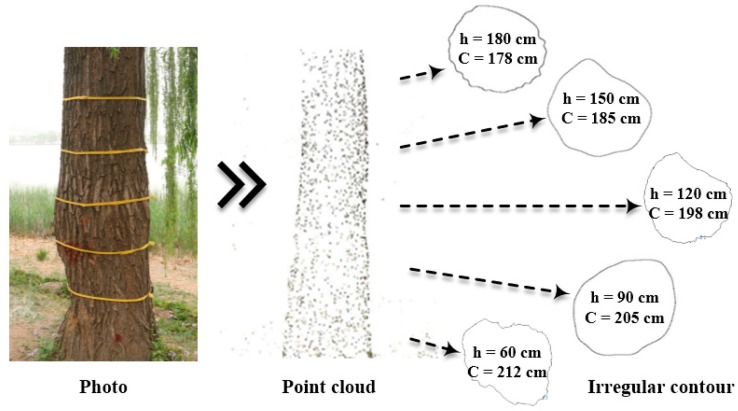
The trunk contour by point cloud method.

Agisoft PhotoScan [34] was used to create point cloud data by taking digital photographs as input collected along the circumference of a fixed radius as the center of the tree trunk. The photographs of the tree in the field, the dense point cloud of the trunk and the outlines of cross-sections are shown in Figure 3. C is the perimeter of the cross-section, and h is the height of the cross-section. The exact outline of the trunk can be observed by the top view and the cross-section of the model. The model containing point cloud data can provide absolute coordinate information for reconstructing trunk cross section with GPR tomography.

### 2.5. Trunk Detection Method

**Figure 4:**
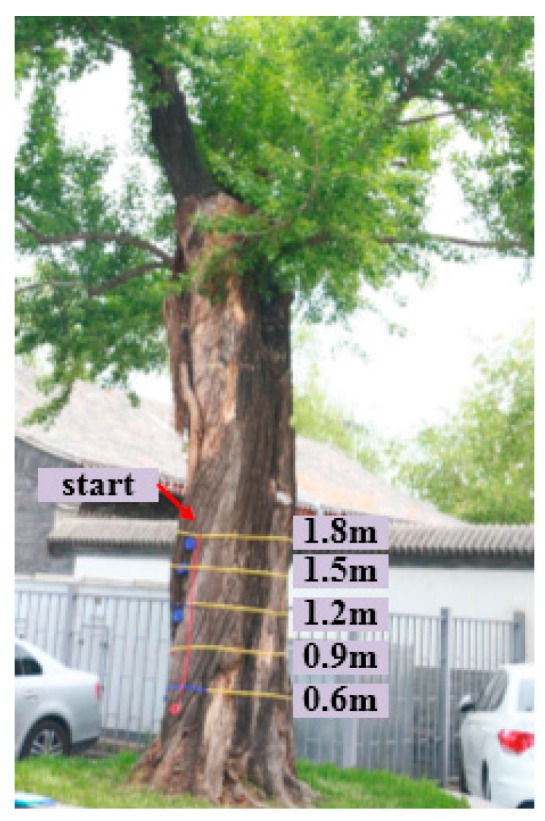
Sketch map of detection.

The tree trunk was detected by scanning multiple circles along the tangent direction of cross-sections. The radar echo was used to analyze the internal structure. The cross sections of five heights (0.6, 0.9, 1.2, 1.5, and 1.8 m) of each tree trunks were detected by the TRU™ system (Figure 4). Five heights and north starting line were signed by Velcro straps. The cross-section circumferences of trunks were measured. Then, the GPR data was collected clockwise from the beginning of the north with constant speed. The radar antenna should stick to the bark. The data was recorded by data collector after rotating one circle. The detection at the same height repeated three times in order to ensure the accuracy. The point cloud data of trunks were collected to construct a 3D model. Irregular outer contours at five heights were obtained according to the mark positions. Finally, the analysis routine was used to analyze the data. The structure and defects of the trunk were obtained.

**Figure 5:**
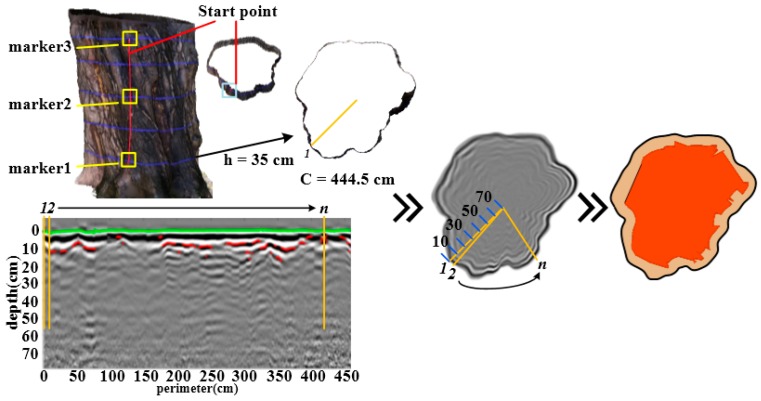
Coordinate mapping of GPR B-Scan.

To analyses measured GPR data, first DZT data were pretreated by zero correction, direct current removal [35], noise filtering and range gain [36], and the effective radar data in the internal structure and anomalous areas of the trunk were obtained. Then, the internal radar data of trunk and point cloud data of the outer contours were processed, respectively. The methods of defect recognition were mainly the Hilbert algorithm, estimation of relative dielectric constants from reflection amplitudes and time delay estimation. Finally, data mapping between trunk internal GPR data and contour data of cross-section was presented considering equal arc segments and coordinate system transformation. As presented in Figure 5, the A-scan models along the yellow line were stitched together to provide a B-scan image.

Ideally, the three-dimensional model is the same as the shape and size of the real tree trunk, but in actual operation, due to data sources and construction methods, some errors will inevitably occur. In order to correct the accuracy of the 3D model, control points of nylon straps are taken as the reference point, as shown in marker 1~3 in Figure 5. Coordinates of the markers derived from the model and the measured coordinates by the total station are converted to the same coordinate system for error correction. The comparison of the coordinates of the comparison point error was used to correct the measured 3D model. The measured contrast error of about 1 cm, which meet the requirements of measurement accuracy.

### 2.6. Test of the GPR Ray-Based Tomography Method

#### 2.6.1. Numerical Simulations

**Figure 6:**
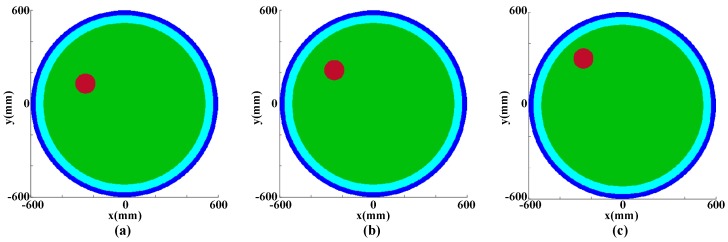
The simulation models of cavities; (**a**) The distances between the cavity center and the interface was 350 mm; (**b**) The distances between the cavity center and the interface was 250 mm; (**c**) The distances between the cavity center and the interface was 150 mm.

For better understanding of the image for a circumferential data acquisition and to verify the validity of the GPR ray-based tomography method mentioned above, circular models of tree trunks with different decay areas were examined using numerical modelling. In particular, gprMax2D, which solves two-dimensional Maxwell’s equations using the Finite-difference Time-Domain (FDTD) method, was used in this study. The source function was Ricker wavelet with the dominated frequency of 900 MHz, time interval was 0.005 ns, and time window was 15 ns. The air layer with a thickness of 30 mm was simulated by the free-space of GprMax. The transmitter was in the air layer, circumferentially surrounding the trunk model to emit electromagnetic wave into the trunk. The transmitter started from the north, increasing 5 mm counterclockwise to the next transmitting position. Figure 6 shows ideal rotted trunk models with three round cavities of different locations in the sapwood layer. The brown areas at a radius of 50 mm were the cavities filled by air with a relative dielectric constant of 1. The distances between the cavity center and the interface were 350, 250 and 150 mm, respectively. The areas at the deep-blue color indicated were the air layers. The areas at the light-blue and green color indicated were the bark layers and the sapwood layers with the thickness of 20 and 550 mm, the relative dielectric constants of 5 and 11, respectively.

#### 2.6.2. Laboratory Measurements

For a comparison with the results of the numerical simulations, the laboratory measurements of samples from the field (see next section) were performed, with the TRU™ radar and analysis routine. Although the samples were not exactly the same as the real tree trunks, it turned out to be a very useful compromise between numerical simulations and real tree trunk measurements. To validate the GPR results, we compared GPR estimations with the actual structure of the sample. There were 15 samples numbered T1–T15, containing cavities in different sizes, with the diameter of 50–70 cm, the height of 120 cm approximately and a water content of 50–70%. The ambient temperature in the laboratory was 26 °Celsius. Repeated measurements were performed to reduce the error. Each sample was analyzed three times and a group of GPR data with better continuity was taken. The results of the analysis routine were compared with that of TreeWin.

#### 2.6.3. Field Experiments

**Figure 7:**
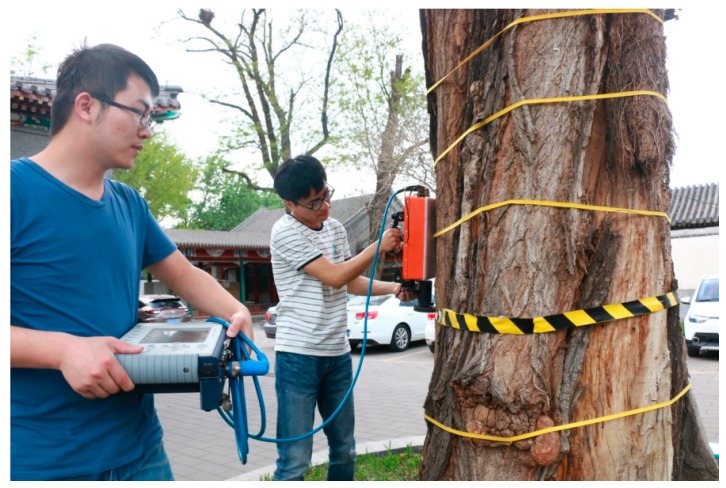
Field experiment.

In order to compare the numerical simulations and the laboratory measurements with the reality, experiments of real tree trunks were performed in the Summer Palace, Beijing, China (Figure 7). The Summer Palace is one of the best preserved royal gardens and plays an important role in historical and cultural heritage. There are 1601 ancient trees in the Summer Palace with various kinds and site conditions, which require professional detection and protection. In order to investigate the health condition of ancient trees, the experiments are performed in the Summer Palace, which can provide the theoretical basis and data support for the conservation of ancient trees in gardens. The same radar equipment and routine as for the laboratory measurements were used.

## 3. Results

### 3.1. Results of Numerical Simulations

**Figure 8:**
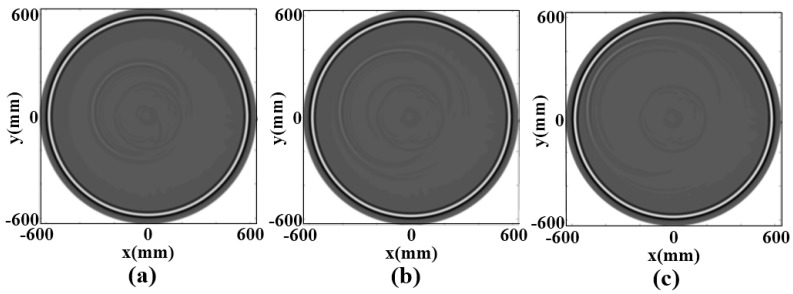
The simulation result of cavities at different thickness; (**a**) The distances between the cavity center and the interface was 350 mm; (**b**) The distances between the cavity center and the interface was 250 mm; (**c**) The distances between the cavity center and the interface was 150 mm.

Figure 8 shows the reconstructed result in numerical experiments. The relative dielectric constants are estimated according to reflection amplitudes. The estimation relative dielectric constant of bark layer is 5.008 on average. The estimation relative dielectric constant of sapwood layer is 11.003 on average. Compared to relative dielectric constants in GprMax, the errors of the predicted values are acceptable. The linear strong reflection signals which are echo images of interfaces between the air and bark layers and between the bark and sapwood layers, and the echo superposition due to closing time, are observed at the travel time of 1–2 ns. There are hyperbolic strong reflection signals at the travel time of 6, 4, and 2 ns, respectively, at the bottom of which there are a number of hyperbolic weak reflection signals, which are produced for circular cavities. The depth range of the hyperbolic vertex is the cavity area The center is at the bottom of the vertex. The occurrence time of echo vertex is related to the location of the cavity. The occurrence time gets small as the distance between the cavity and the bark layer decreases. Similarly, the energy of the radar reflected wave becomes stronger with the smaller the distance from the bark layer.

### 3.2. Result of Laboratory Measurements

**Figure 9:**
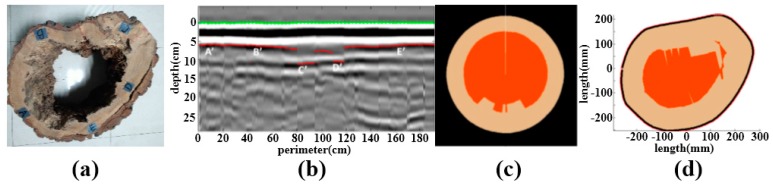
(**a**) Samples of T1; (**b**) B-scan data with the defect region boundary expressed in the red line and point A'B'C'D'E'; (**c**) The results of TreeWin; (**d**) The results of the analysis routine

One of the results in laboratory measurements, sample T1, is shown in Figure 9. The orange area in the center and the light pink area are respectively the cavity and the healthy trunk. The black curve is the trunk outline. The area errors of T1, T2, and T3 are shown in Table 1.

As noted in Figure 9, B-scan data cannot observe the internal structure of trunk directly, while it is direct and clear that the two-dimensional analysis images obtained by the analysis routine can show the location and shape of anomaly area and the outline of the trunk. It can be found that the results of TreeWin are quite different from that of samples, while the area error rate of the analysis routine is less than 5%. It has a satisfactory accuracy and visualization for the internal anomaly detection of tree trunks by analysis routine.

**Figure 10:**
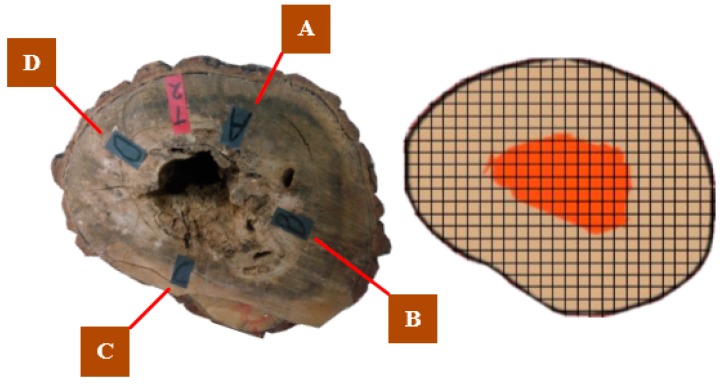
The thickness and area measure of samples.

The thicknesses of A, B, C, D directions between bark and anomaly and areas are used to testify the accuracy, shown in Figure 10. The thicknesses of four directions are measured by vernier caliper. The areas of cross-sections and anomalies are calculated by the grid method. The relative errors of thickness and area are shown in Table 2 as an example.

In Table 2, $\bar{L_{A}}$, $\bar{L_{B}}$, $\bar{L_{C}}$ and $\bar{L_{D}}$ are the thickness of A, B, C, D directions, respectively. $\bar{S_{1}}$ and $\bar{S_{2}}$ are the cross-sectional area and anomaly area. $\bar{T_{M}}$ represents measure value. $\bar{T_{D}}$ represents detection value. As for sample t1, the error of thickness is 7.33%. The error of cross-sectional area and anomaly area are 1.19% and 3.23%, respectively. Among 15 tested samples, the average error of thickness is 7.98%, with the range of [6.10, 9.04]. The average error of cross-sectional area is 3.37%, with the range of [1.19, 4.87]. The average error of anomaly area is 3.62%, with the range of [1.86, 4.87].

### 3.3. Result of Field Experiments

**Figure 11:**
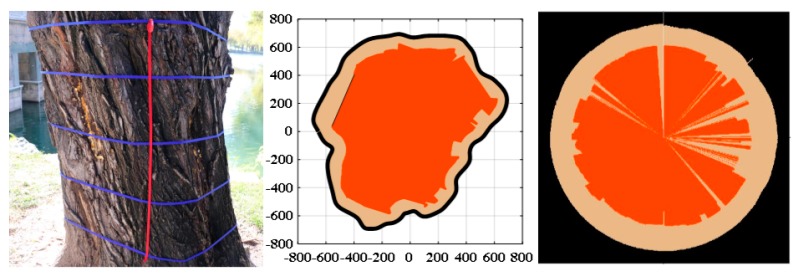
Detection result of tree L01 in the Summer Palace.

The result of one willow tree L01 located beside the western shore of Kunming Lake in the Summer Palace is shown in Figure 11. It can be observed that the detection result and imaging identification obtained by the analysis routine is more similar to the actual situation than the TreeWin. The percentages of the cross-section defect area in the section area at each height are shown in Table 3. There are more serious cavities inside the tree trunks. The decays and cavities inside the trunks increase as the age of tree growth. In addition, plant diseases and insect pests are also important factors for the abnormal growth of tree trunks, such as longicorn and dry rot fungus.

**Figure 12:**
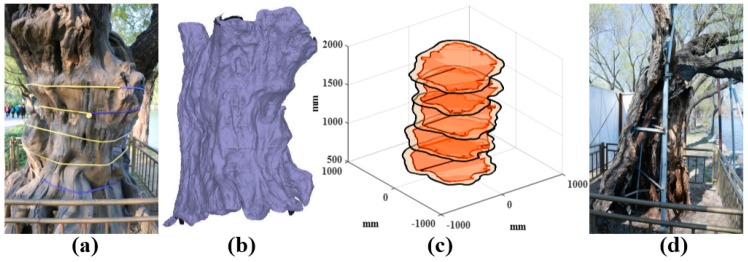
The detection result of tree L05 in the Summer Palace. (**a**) The willow tree in the field; (**b**) The trunk reconfiguration model; (**c**) The 3D analysis result; (**d**) The rejuvenation in order to compare with the detection results.

The protection of ancient trees is always dependent on the experience of experts. It is difficult to accurately identify its internal growth status, as most erosion is started from the inside of the trunk. A lot of trees have been rejuvenated in the Summer Palace (e.g., L05, shown in Figure 12a), and the internal defects are hardly seen from the exterior. The trunk reconfiguration model of L05 is shown in Figure 12b. The 3D analysis result is shown in Figure 12c. The vertical axis represents the height of the trunk, and the orange areas represent cavities. The 3D result is more intuitive.

Figure 12d shows the rejuvenation of L05. It can be observed that the inside of the trunks is almost empty, which are supported only by iron stents and ceramsite. The external bark is incomplete, and needs to be repaired with false barks. It is verified that GPR technology can detect the anomaly inside the trunk non-invasively by comparing the detection results and the actual situations in rejuvenation. The method provides a theoretical basis for the health assessment and rejuvenation of the trunk.

## 4. Discussion

Accurate tree trunk structure estimation is an important issue to prevent collapses of trees in urban areas or around roads. The destructive inspection of core drilling or in an extreme case chopping down of the tree cannot apply to living trees. GPR represents a prominent alternative since it can be used for nondestructive trunk profiling. The GPR system has an excellent ability to reconstruct the internal structure of a living trunk cross-section. Hilbert algorithm allows for high accurate characterizations. The point cloud technique provides the trunk high-precision coordinate information which directly affects the accuracy of final tomography results. The layer localization combined with the contour information from point cloud data permitted high-resolution visualization of the irregular shape of trees. It also improved the accuracy of the anomaly detections within the trees. Furthermore, the 3D structure consisting of five cross-sections is reconstructed.

It is very important to know the propagation path of the electromagnetic waves for determining the position, shape and size of an observed target inside a circular trunk. The internal structure of trunks plotted according to the analysis routine fitted very well the structure of the numerical simulations and also of laboratory measurements. In laboratory measurements, the detected thicknesses of trunk layers were consistent with the measured values, which showed an acceptable effect in contrast detection. In field experiments, the comparison between observed results from field rejuvenation and predicted results from GPR suggested a satisfactory accuracy, which could satisfy the requirements of tree trunk detection non-invasively.

## 5. Conclusions

In this study, the GPR ray-based tomography technique was proposed as an internal structure estimation method for tree trunks based on their irregular contours. It is very important to know the analytical description of the curve for determining the location, shape and size of anomalies inside the tree trunk. The reflection curves of anomalies and interfaces between different layers were observed in numerical simulations. It can help to make a better prediction of the positions of holes and decays in tree trunks in future. Moreover, this study highlighted the importance of the use of a point cloud technique for reconstructing the internal structure of tree trunks to obtain accurate tomography images. The laboratory measurements showed that the applied routine rendered more promising results than TreeWin.

The results showed that predicted errors of thickness and area were less than 10% and 5%, respectively, which could satisfy the requirements of real tree trunk detection. From the real tree trunk measurements, it could conclude that most of the reflections were naturally more complex than in numerical and laboratory images. The 3D analysis results show that the predicted internal anomalies are consistent with the rejuvenation situation. Our work indicates that the GPR ray-based tomography technique can be used as a fast, non-invasive method in the internal structure estimation of tree trunks, and has great potential in the health assessment for ancient trees.

## Figures and Tables

**Table 1:** Errors of detection results.

No.	Water Content	Actual Area/cm^2^	Detection Area/cm^2^	Error/%
T1	42.5~85.4%	625.30	613.64	1.91
T2	40.3~89.8%	502.15	518.35	3.23
T3	35.8~53.6%	109.24	104.15	4.66

**Table 2:** Relative error of sample T1.

	$\bar{L_{A}}/\mathrm{cm}$	$\bar{L_{B}}/\mathrm{cm}$	$\bar{L_{C}}/\mathrm{cm}$	$\bar{L_{D}}/\mathrm{cm}$	$\bar{S_{1}}/{\mathrm{cm}}^{2}$	$\bar{S_{2}}/{\mathrm{cm}}^{2}$
$\bar{T_{M}}$	14.53	8.33	6.56	10.75	751.30	502.15
$\bar{T_{D}}$	15.16	7.45	7.14	11.35	760.21	518.35
error/%	7.33				1.19	3.23

**Table 3:** **Table** **3.** Field experiment results.

No.	Average Perimeter of Cross-Section/m	Ratio of Abnormal Area at Different Heights				
		0.6	0.9	1.2	1.5	1.8
L01	4.35	65.71%	70.31%	68.75%	74.19%	73.53%
L02	2.74	60.32%	54.37%	55.32%	63.16%	⁄
L03	4.91	68.89%	65.79%	74.68%	69.89%	⁄
L04	5.02	71.74%	75.61%	76.92%	76.60%	⁄
L05	4.91	71.68%	73.68%	72.22%	68.42%	⁄
L06	4.89	62.33%	66.67%	58.93%	57.14%	⁄
L07	3.66	58.12	53.19	58.91	50.83	52.65
L08	1.81	59.26	51.85	69.77	59.20	59.86
L09	1.95	67.28	56.15	53.94	62.94	69.14
L10	2.17	60.15	61.75	62.68	59.42	52.75
